# Schwannoma causing resorption of zygomatic arch

**DOI:** 10.4103/0973-029X.80020

**Published:** 2011

**Authors:** Ajaz A Shah, Suhail Latoo, Irshad Ahmad, Altaf H Malik, Amrit Pal Singh, Shahid Hassan

**Affiliations:** *Department of Oral and Maxillofacial Surgery, Government Dental College, Srinagar, India*; 1 *Department of Oral and Maxillofacial Pathology, Government Dental College, Srinagar, India*

**Keywords:** Bone, erosion, mandible, neurilemmoma, schwannoma

## Abstract

Schwannoma (also known as neurilemmoma, peripheral glioma and peripheral nerve sheath tumor) is a common, histologically distinctive, benign, usually encapsulated, peripheral nerve tumor of Schwann cell origin. Schwannomas can appear anywhere in the body, but are more frequently reported in the head and neck with an incidence of 25–48% in maxillofacial region. Resorption of bones due to schwannoma is rarely noticed in maxillofacial region. We hereby present a case report of schwannoma in a 35–year-old female, causing resorption of zygomatic arch along with review of literature.

## INTRODUCTION

Schwannoma (also known as neurilemmoma, neurolemoma, neurinoma, perineural fibroblastoma, peripheral glioma and peripheral nerve sheath tumor) is a slow-growing, benign neoplasm derived from Schwann cells, which are sheath cells that cover myelinated nerve fibers. Schwannomas may be encapsulated and can appear anywhere in the body, but are more frequently located in the head and neck.[1–5] Most commonly, the tumor appears in the paravertebral region of the retroperitoneum, pelvis, mediastinum, extremities, nasal cavity, nasopharynx, orbit, parapharyngeal space, larynx and oral cavity.[6] Intraoral development is uncommon (only 1%). In this area, in a decreasing order of frequency, the mobile portion of the tongue, the palate, the cheek mucosa, the lip and gingiva are the most frequent locations.[7] In the tongue, the tip is the least affected part.[8–12] This lesion has been widely reported, but it was only hypothesized that one of the mechanisms by which it involves the bone is through secondary erosion from a soft tissue or periosteal tumor.[13–15] Resorption of bones due to schwannoma is rarely noticed in maxillofacial region. We hereby present a case report of schwannoma in a 35-year-old female, causing resorption of zygomatic arch.

## CASE REPORT

**Figure 1:**
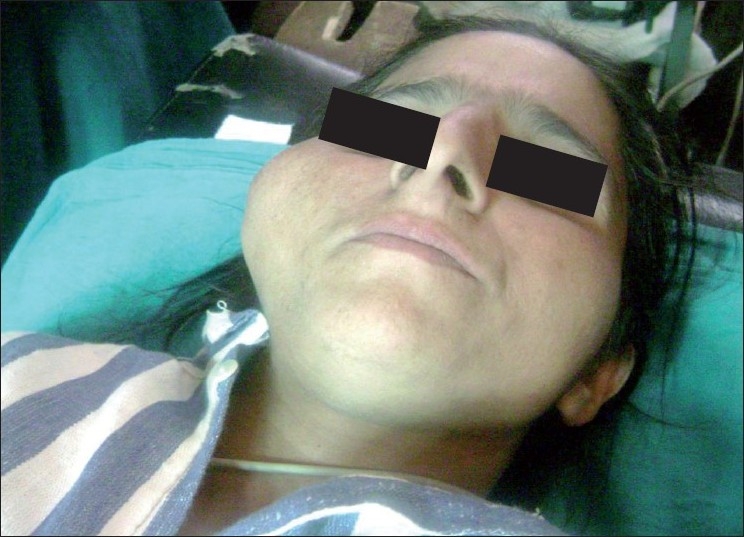
A firm mass measuring 5 × 3 × 2 cm in size in the right zygomatic region

A 35-year-old female presented at our department complaining of swelling in right facial region since 2 years. There was no history of pain, paresthesia or limitation in mouth opening. During the examination, a firm mass, measuring 5 × 3 × 2 cm in size, was observed in right zygomatic region. The mass was bound to a part of zygoma [Figure 1].

**Figure 2:**
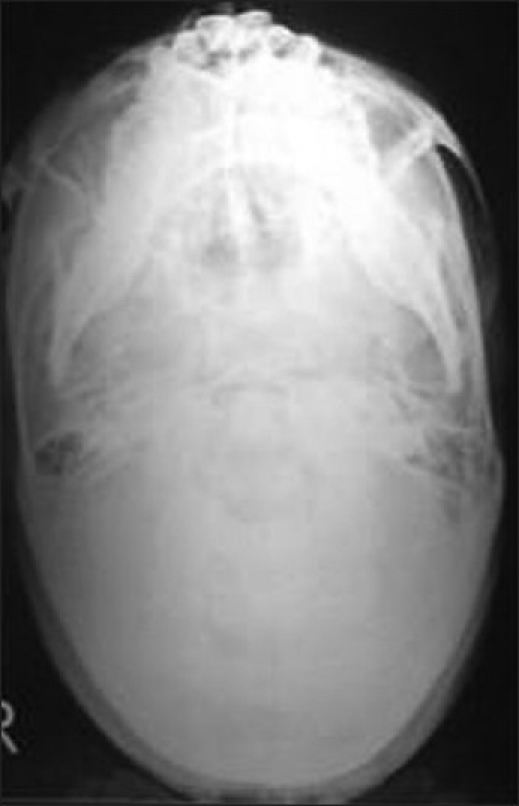
A submentovertex view of skull showed resorption of zygomatic arch

**Figure 3:**
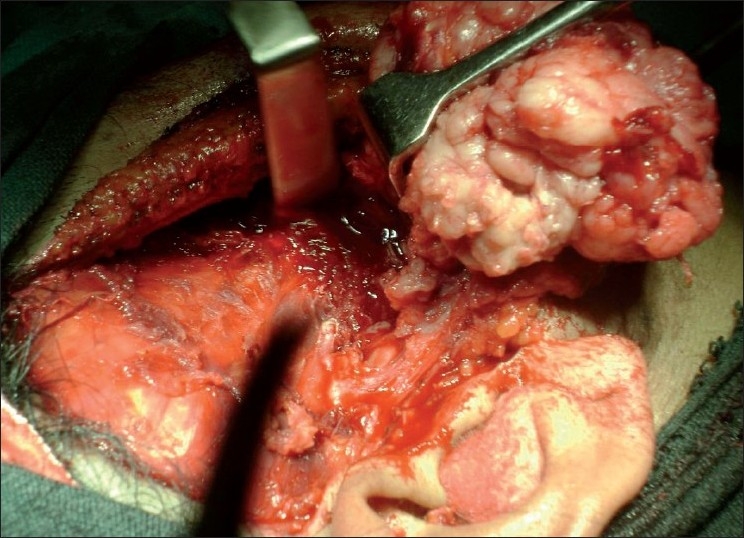
Isolation of tumor mass

**Figure 4:**
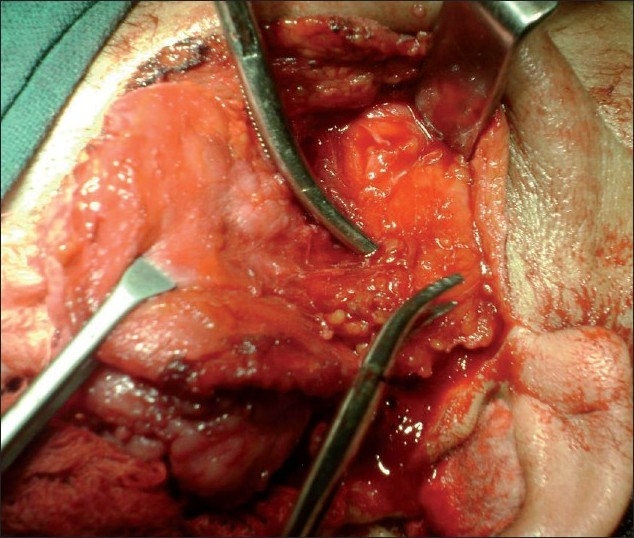
Erosion of zygomatic arch

A submentovertex view of skull showed resorption of zygomatic arch [Figure 2]. Under general anesthesia, Alkayet Brameley incision was placed, and a round, pale yellow encapsulated mass at the zygomatic arch was identified. The mass was removed *in toto* [Figure 3]. Beneath the mass, we noticed a secondary erosion of the zygomatic arch [Figure 4]. During the surgical removal, we could not identify the nerve from which the tumor derived. There was no relationship between the tumor and the underlying indented bone.

**Figure 5:**
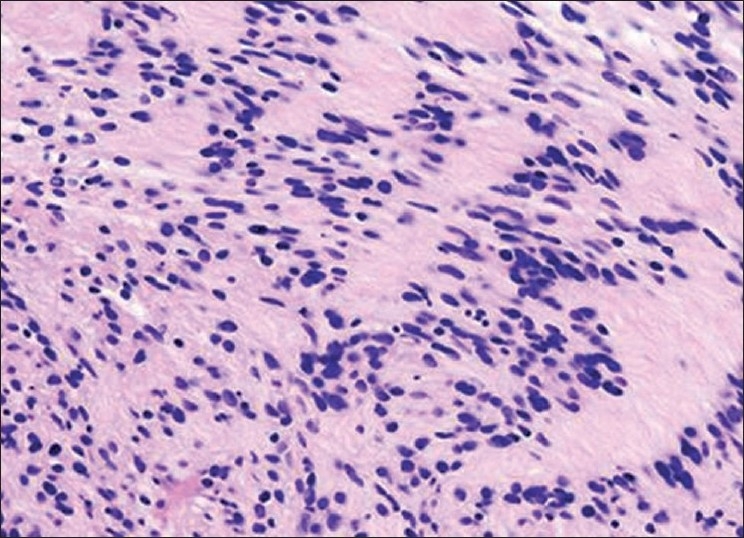
Histopathologic picture showing interlacing fascicles of compact spindle cells with twisted nuclei. The nuclear palisading formed the Verocay bodies

An oval, sharply demarcated, encapsulated, firm nodule measuring 3 cm in diameter was submitted for histopathologic examination. The cut surface was yellowish white and smooth. Microscopic analysis revealed that the tumor mass was composed of interlacing fascicles of compact spindle cells with twisted nuclei [Figure 5]. The nuclear palisading formed the Verocay bodies [Figure 5]. A fibrous capsule surrounded the tumor nodule. On the basis of clinical, radiological and histopathologic findings, final diagnosis of schwannoma was made. Patient is on 6-monthly follow-up since 1 year, and till date, no signs of recurrence have been noticed.

## DISCUSSION

Oral and maxillofacial peripheral nerve tumors include schwannoma, neurofibroma, nerve sheath myxoma, palisaded encapsulated neurinoma, mucosal neurinoma associated with multiple endocrine neoplasia III, traumatic neuroma and granular cell tumor.[16]

There are three mechanisms by which schwannomas may involve a bone: (1) a tumor may arise centrally within a bone, (2) a tumor may arise within a nutrient canal and produce canal enlargement, or (3) a soft tissue or periosteal tumor may cause secondary erosion and penetration into a bone.[13–15] This case demonstrates an example of schwannoma secondarily involving a bone.

Schwannomas most often occur in the fourth and fifth decade of life with a 1.6:1 female predilection. The duration of symptoms varies from a few months to a few years. A majority of these tumors have a long duration because of their lack of symptoms and slow growth.

A review of the English literature revealed three cases similar to ours with regard to clinical and radiological features. In 1954, Bruce[17] described a tumor which was located on the left edentulous mandibular alveolus. Dental radiographs revealed some osteolysis in the mandibular alveolus subjacent to the tumor. Surgical excision and enucleation of the tumor left a smooth concavity in the body of the mandible, which was not associated with the mandibular canal or nerve, but histologically it was neurofibroma. Worth[18] describes neural sheath tumors that arise subperiosteally causing saucerization of the bone. These tumors are radioluscent and may or may not have a cortical outline. One such neurofibroma was reported by Schneider *et al*.[19] The central portion of the lesion was described as mottled and, at surgery, the lesion appeared to be covered with bone. Mortada[20] and Sciubba and Sachs[21] reported cases of schwannomas with secondary penetration into the bone, but they could not determine if the lesion arose centrally or from the periosteum. Kun *et al*.[22] reported that preoperative diagnosis was correct in only 4 out of 49 cases in their study. They concluded that it was difficult to make a confirmed diagnosis on the basis of imaging findings.

Radiographically, schwannoma is commonly unilocular and associated with bone resorption.[23] It may resemble many benign conditions such as odontogenic or periodontal cyst, ameloblastoma, angioma and benign odontogenic tumor. If the tumor is large with destruction of cortical bone, it may resemble a malignant lesion.[24] Some schwannomas have reportedly turned malignant[25,26] and mandibular malignant schwannoma has been reported.[27] When degenerative changes are very pronounced, calcifications, hyalinizations, hemorrhages and atypical nuclei, as well as cystic formations will appear, but these changes do not lead to malignancy.[28,29] The preoperative diagnosis of schwannoma is rare,[28] although with digital intravenous subtraction angiography, computed tomography (CT) scans and magnetic resonance imaging (MRI), the probabilities are increased.[30] Magnetic resonance patterns for neurofibromas have the following characteristics: low to intermediate signal intensity on T_1_-weighted images, enhancement of the solid component of the tumor after administration of contrast medium, heterogeneity on T_2_-weighted images, and multiple target signs due to a central collagen area (some patients).[31] MRI findings of intraosseous schwannoma of the mandible help in differentiating solid from purely cystic lesions (e.g., dentigerous cysts, periodontal cysts).[32] Yamazaki *et al*.[33] reported that ultrasound and MRI were effective in preoperative imaging diagnosis of schwannoma originating in the mental nerve. They also suggested that the compatibility of photographic parameters in MRI techniques for identifying nerves, particularly the final branch with a short diameter in the extracranial region, requires careful discussion in the future. On MRI, a tumor can be delineated as solid, cystic, or mixed, based on its pathological characteristics. Since the imaging findings are variable, it is difficult to arrive at a confirmed diagnosis based only on such findings. In this case, malignancy could not be completely ruled out by preoperative imaging findings. Wakoh *et al*.[34] reported two cases of schwannoma displaying marked cystic changes, one in the temporalis muscle and one in the submandibular space. They concluded that MRI should depict the nerves and allow identification of the origin of schwannoma. MRI can show not only the tumor and the capsule, but also in certain cases the nerve from which it has developed.[35] Only a few cases of schwannoma in the oral floor have been reported. However, when the characteristic findings are observed on CT and MRI, schwannoma should be added to the differential diagnosis.[36] In the case presented by Wakoh *et al*., based on the pre-operative CT and MRI findings, a malignant tumor derived from the sublingual gland was suspected. Intraoperatively, adhesion of the mass to circumferential regions was not observed, but nerves penetrated into the mass at several places. Based on operative findings, the mass was thought to be a tumor derived from the lingual nerve. Almeyda *et al*.[37] reported a case of submandibular schwannoma (3.5 cm × 2 cm × 2 cm) misdiagnosed preoperatively. The differential diagnosis of adenolymphoma (Warthin’s tumor) was based on the clinical examination, ultrasound and two fine needle aspirations. Intraoperatively, the surgeon noted adherence of the tumor to a branch of the lingual nerve. The mass and submandibular gland were excised “*en bloc*”. Even with advancement in imaging techniques, the diagnostic dilemma remains.[37] MRI has been of greater use, with a distinctive target pattern demonstrated by most, but not all, schwannomas.[38] In the case presented by Asaumi *et al*.,[39] the ultrasound, CT and MRI appearance of schwannoma of the upper lip (3.8 cm × 1.8 cm × 1.4 cm) correlated well with the histological features. MRI was particularly helpful in showing the internal characteristics of the encapsulated mass. They concluded that because most tumors of the upper lip present as relatively small lesions, establishing the differential diagnosis using ultrasound, CT and MRI should not be considered as routine or necessary. We completely agree with this attitude because in our case, the tumor measured 3 cm in diameter.

Although schwannomas originate from the nerve tissue, locating the nerve of origin exactly can be impossible. Direct relation with a nerve can be demonstrated in only approximately 10–50% of the cases.[7,28,40–43] Tumors arising from the small nerves are freely mobile, but mobility is restricted along the axis in those arising from large nerves.[44] The growth of these lesions will cause displacement and compression of the surrounding normal nerve tissue.[12] Yamazaki *et al*.[33] stated that the lesions are diagnosed as peripheral nerve sheath tumor when the tumor is connected directly to the nerve, even though the nerve itself cannot be identified. However, in their presented case, the possibility of malignancy could not be ruled out from the preoperative imaging findings and the clinical course. The nerve of origin is often not identified at the time of surgical excision, although if presented, it is displaced to the side by the expanding tumor.[37] Arda *et al*.[45] presented a schwannoma arising from the parasympathetic fibers of the lingual nerve. They found only a few nerve fibers, which were thought to be the parasympathetic nerve of the sublingual gland, attached to the tumor. The results of CT imaging did not help them preoperatively for the diagnosis of the mass and fine needle aspiration biopsy was not useful.

For the differential diagnosis, neurofibroma, granular cell tumors, lipoma, fibroma, leiomyoma, rhabdomyoma, nerve sheath myxoma, adenoma, neuroma, granular cell tumor, neurothekeoma and perineurioma should be considered.[8,31,46,47] The differentiation between schwannoma and neurofibroma is essential because an apparently “solitary” neurofibroma may be a manifestation of neurofibromatosis. Around 15–16% of patients with neurofibromatosis will present with malignant transformation in one or more lesions, contrary to schwannoma.[48] The recurrence rate of a schwannoma is lower than that of a neurofibroma because of encapsulation.[49] The differentiation between these two neoplasms is imperative because neurofibromas tend to recur frequently and have the potential for malignant transformation. It is difficult, however, to differentiate an intraosseous schwannoma from an ameloblastoma associated with a substantial solid component.[50] A small and slow-growing mass in the tongue with a positive history of tongue bite is first suggestive of schwannoma, as well as neurofibroma, lingual cyst and minor salivary gland tumor.[51] Neurofibromas lack the thick collagenous capsule of schwannomas and instead are surrounded by a variably thickened perineurium and epineurium. Neurofibromas also lack the Antoni Type A and B patterns and Verocay bodies typical of schwannomas. Immunoreactivity for S-100 protein is observed in only a portion of the cells comprising a neurofibroma, as opposed to uniform reactivity throughout a schwannoma.[5] Neurofibroma is generally non-encapsulated and lobulated with an irregular surface and, unlike the schwannoma which pushes away the associated nerve, it becomes intertwined with the nerve of origin.[28–30] Neurofibroma is difficult to remove; it recurs or persists when resection is incomplete, and in cases of neurofibromatosis, it can transform into a malignant tumor.[29] Malignant transformation of schwannoma is in contrast to neurofibroma, an exceptionally rare event and for practical purposes can be discounted.[52]

In conclusion, we have reported here a rare case of schwannoma with secondary erosion of the zygomatic arch. The tumor may have originated from a branch of infraorbital nerve and may have extended into the zygomatic arch, creating a bony defect.
